# Supplementary material for: Clove Oil-Nanostructured Lipid Carriers: A Platform of Herbal Anesthetics in Whiteleg Shrimp (Penaeus vannamei)
Source: Foods. 2022 Oct 11;11(20):3162. doi: 10.3390/foods11203162 (PMC9602023; doi:10.3390/foods11203162)
Supplement: Supplementary file 1 [file foods-11-03162-s001.zip › foods-1896598-supplementary.pdf]

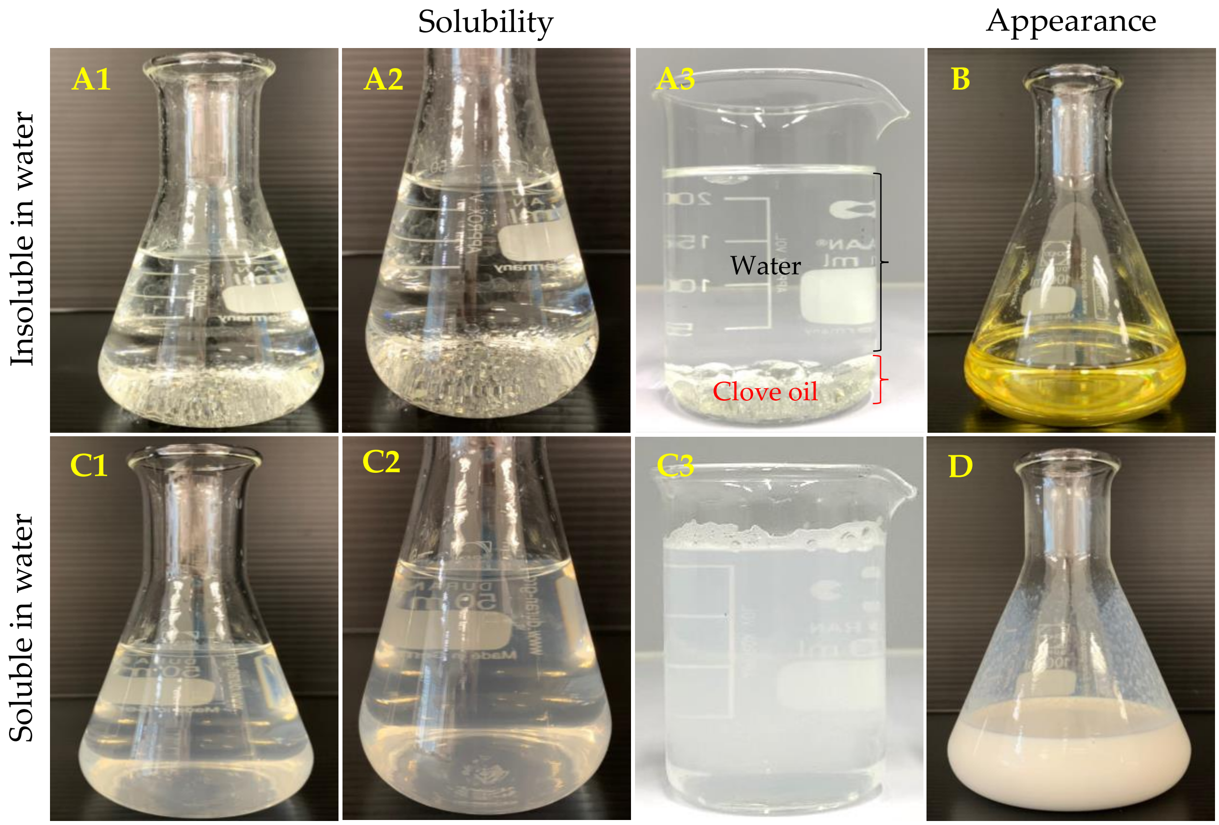

Figure S1. Solubility of STD clove oil (A1-3) and clove oil NLCs (C1-3). The appearance of STD clove oil (B) and clove oil NLCs (D).

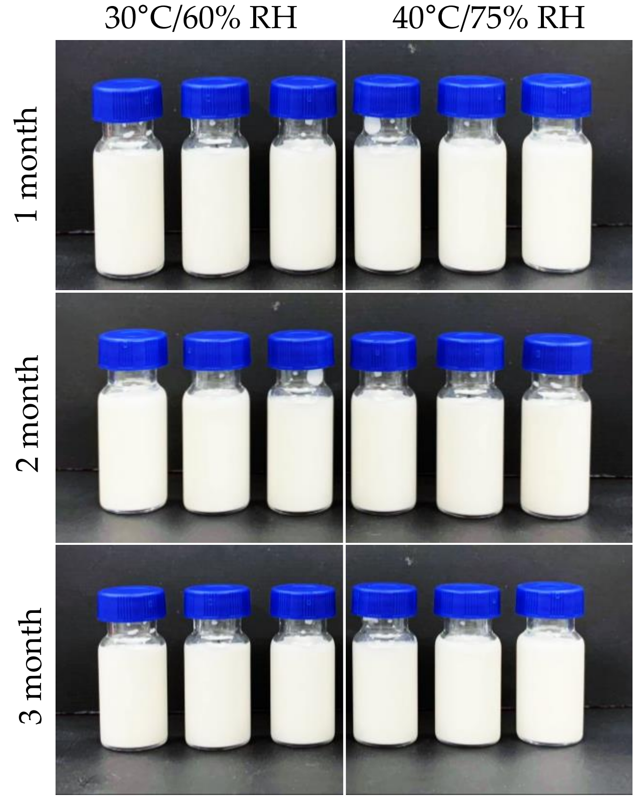

Figure S2. Stability of clove oil NLCs in different conditions (30°C/60% RH and 40°C/75% RH) after storage of 1, 2 and 3 months, respectively.
